# Notes for authors

**DOI:** 10.1155/S1463924685000050

**Published:** 1985


### References

1. HUMPHREY, A. E., *Process Biochemistry*, **12** (1977), 19.
2. COONEY, C. L., WANG, H. and WANG, D. I. C., *Biotechnology and Bioengineering*, **18** (1976).
3. CARLBERG, G., HOLMBERG, A. and SIEVÄNEN, R., *Fermentation of Bac. thüringiensis for exotoxin production – A Process analysis study.* (Helsinki University of Technology, Systems Theory Laboratory, Report B51,(1978).
4. MOR, J.-R., ZIMMERLY, A. and FIECHTER, A., *Analytical Biochemistry*, **52** (1974), 614.
5. ZABRISKIE, D. W. and HUMPHREY, A. E., *Biotechnology and Bioengineering*, **20** (1978), 1295.
6. ROY, R. B. and BUCCAFURI, A., *Journal of Automatic Chemistry*, **2** (1980), 159.
7. HÖGYE, I. and NAGY, CS., *Proceedings 5th Fermentation in Colloquium* (Debrecen, Hungary, 1981), 184.
8. BENKE, J., Automatic assay for L-tryptophan determination in fermentation broth. (B. Sc. Thesis, University of Technical Sciences, Budapest, 1981).
9. DEAN, J. R. and STEWART, R. B., *Journal of Automatic Chemistry*, **1** (1979), 252.
10. OJAMO, H. and LEISOLA, M., *27th IUPAC Congress* (Helsinki, 1979), 575.
11. LEISOLA, M., OJAMO, H. and KAUPPINEN, V., *Enzyme Microbiological Technology*, **1** (1979), 51.
12. LEISOLA, M., OJAMO, H., KAUPPINEN, V., LINKO, M. and VIRKKUNEN, J., *Enzyme Microbiological Technology*, **2** (1980), 121.
13. LEISOLA, M. and KAUPPINEN, V., *Biotechnology and Bioengineering*, **20** (1978), 837.
14. KUHLMANN, W., MEYER, H. D. and SCHÜGERL, K., *Biotechnologie und Verfahrenstechnik*, **16** (1982), 518.
15. HILL, F. F. and THOMMEL, J., *Process Biochemistry*, **17** (1980), 16.
16. Standardized assays for water investigation. Chemical methods. COMECON Standards (VITUKI, Budapest, 1968).
17. NYESTE, L., SZIGETI, L., VERES, A., PUNGOR, E. Jr., KURUCZ, I. and HOLLÓ, J., *Biotechnology and Bioengineering*, **23** (1981), 405.
18. ÅSTRÖM, K. I., *Introduction to Stochastic Control Theory* (Academic Press, New York, 1970).
19. KEVICZKY, L., New Adaptive Discrete MV controllers. D.Sc. Dissertation (Budapest, 1979).
20. KEVICZKY, L. and HETTHÉSSY, J., *Periodica Polytechnica*, **18** (1974), 291.
21. PIRT, S. J., *Principles of Microbe and Cell Cultivation* (Blackwell, Oxford, 1975).
22. MARTIN, J. F., Control of antibiotic synthesis by phosphate. In GHOSE, T. K., FIETCHER, A. and BLAKEBROUGH, N., *Advances in Biochemical Engineering* **6**, 105 (Springer Verlag, Berlin, 1977).
23. VINCZE, I., *Mathematical Statistics with Industrial Applications* (Müszaki Könyvkiadó, Budapest, 1968).

### PRODUCT DESIGN ASSURANCE IN ENGINEERING

The Society of Environmental Engineers' 11–13 June 1985 conference at Wembley will cover Specifications, Techniques and Case studies.

In conjunction with the conference there will be a major exhibition of environmental equipment.

*Details from Mrs Helen Gibbons, Society of Environmental Engineers Secretariat, Owles Hall, Buntingford, Hertfordshire, UK. Tel.: 0763 71209.*

### NOTES FOR AUTHORS

*Journal of Automatic Chemistry* covers all aspects of automation and mechanization in analytical, clinical and industrial environments. The *Journal* publishes original research papers; short communications on innovations, techniques and instrumentation, or current research in progress; reports on recent commercial developments; and meeting reports, book reviews and information on forthcoming events. All research papers are refereed.

### Manuscripts

Two copies of articles should be submitted to the Editor. All articles should be typed in double spacing with ample margins, on one side of the paper only. The following items should be sent: (1) a title-page including a brief and informative title, avoiding the word 'new' and its synonyms; a full list of authors with their affiliations and full addresses; (2) an abstract of about 250 words – this should succinctly describe the scope of the contribution and highlight significant findings or innovations; (3) the main text; (4) appendices (if any); (5) references; (6) tables, each table on a separate sheet and accompanied by a caption; (7) illustrations (diagrams, drawings and photographs) numbered in a single sequence from 1 upwards and with the author's name on the back of every illustration; captions to illustrations should be typed on a separate sheet. Papers are accepted for publication on condition that they have been submitted only to *Journal of Automatic Chemistry.*

### References

References should be indicated in the text by numbers following the author's name, i.e. Skeggs [6]. In the reference section they should be arranged thus:

to a journal

MANKA, D. P., *Journal of Automatic Chemistry*, **3** (1981), 119.

to a book

MALMSTADT, H. V., in *Topics in Automatic Chemistry*, Ed. Stockwell, P. B. and Foreman, J. K. (Horwood, Chichester, 1978), p. 68.

### Illustrations

Original copies of diagrams and drawings should be supplied, and should be drawn to be suitable for reduction to the page or column width of the *Journal*, i.e. to 85 mm or 179 mm, with special attention to lettering size. Photographs may be sent as glossy prints or as negatives.

### Proofs and offprints

The principal or corresponding author will be sent galley proofs for checking and will receive 50 offprints free of charge. Additional offprints may be ordered on a form which accompanies the proofs.

*Manuscripts should be sent to either Dr P. B. Stockwell or Ms M. R. Stewart, see p. 7.*

